# Effect of Lipid Nanoparticle Physico-Chemical Properties and Composition on Their Interaction with the Immune System

**DOI:** 10.3390/pharmaceutics16121521

**Published:** 2024-11-26

**Authors:** Laura Catenacci, Rachele Rossi, Francesca Sechi, Daniela Buonocore, Milena Sorrenti, Sara Perteghella, Marco Peviani, Maria Cristina Bonferoni

**Affiliations:** 1 Department of Drug Sciences, University of Pavia, 27100 Pavia, Italy; laura.catenacci@unipv.it (L.C.); rachele.rossi01@universitadipavia.it (R.R.); francesca.sechi01@universitadipavia.it (F.S.); milena.sorrenti@unipv.it (M.S.); mariacristina.bonferoni@unipv.it (M.C.B.); 2 Department of Biology and Biotechnology “L. Spallanzani”, University of Pavia, 27100 Pavia, Italy; daniela.buonocore@unipv.it

**Keywords:** lipid nanoparticles, immune response, physico-chemical properties, helper lipids, protein corona, ionizable cationic lipids

## Abstract

Lipid nanoparticles (LNPs) have shown promise as a delivery system for nucleic acid-based therapeutics, including DNA, siRNA, and mRNA vaccines. The immune system plays a critical role in the response to these nanocarriers, with innate immune cells initiating an early response and adaptive immune cells mediating a more specific reaction, sometimes leading to potential adverse effects. Recent studies have shown that the innate immune response to LNPs is mediated by Toll-like receptors (TLRs) and other pattern recognition receptors (PRRs), which recognize the lipid components of the nanoparticles. This recognition can trigger the activation of inflammatory pathways and the production of cytokines and chemokines, leading to potential adverse effects such as fever, inflammation, and pain at the injection site. On the other hand, the adaptive immune response to LNPs appears to be primarily directed against the protein encoded by the mRNA cargo, with little evidence of an ongoing adaptive immune response to the components of the LNP itself. Understanding the relationship between LNPs and the immune system is critical for the development of safe and effective nucleic acid-based delivery systems. In fact, targeting the immune system is essential to develop effective vaccines, as well as therapies against cancer or infections. There is a lack of research in the literature that has systematically studied the factors that influence the interaction between LNPs and the immune system and further research is needed to better elucidate the mechanisms underlying the immune response to LNPs. In this review, we discuss LNPs’ composition, physico-chemical properties, such as size, shape, and surface charge, and the protein corona formation which can affect the reactivity of the immune system, thus providing a guide for the research on new formulations that could gain a favorable efficacy/safety profile.

## 1. Introduction

**Figure 1:**
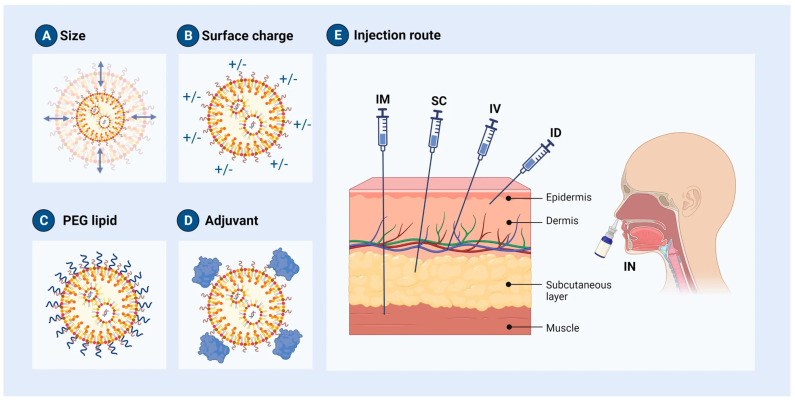
Even slight modifications can change the properties of an LNP. (**A**) By modifying the molar ratio of PEG or the preparation parameters, it is possible to change the LNP size. (**B**) The surface charge of the LNP can be modified by replacing or adding phospholipids to a charged lipid. (**C**) Different PEG–lipid conjugates can be obtained, such as modifying the PEG molecular weight, to influence LNP size, zeta potential, and stability. (**D**) Adjuvants can be added to the formulation to enhance the immune reaction for LNP-based mRNA vaccines. (**E**) There are several methods for administering LNPs, including intravenous (IV), intramuscular (IM), intradermal (ID), subcutaneous (SC), and intranasal (IN). An appropriate route of administration must be determined based on an understanding of the anatomy of the inoculation site and the induced immune action. Reprinted with permission from [4].

Over the past decade, significant progress has been made in understanding the potential toxicity of nanocarriers and how to engineer them to minimize any harmful effects while still utilizing their unique properties. Researchers have found that the toxicity of nanoparticles (NPs) can depend on various intrinsic features, including their size, shape, and surface chemistry, as well as external factors, such as the route of administration (Figure 1). Extensive research has focused on the physico-chemical properties of lipid nanoparticles (LNPs) as they determine the interaction with proteins in biological fluids and with immune cells. By carefully designing and modifying these properties of NPs, it is possible to avoid triggering an immune response or, alternatively, to specifically target the immune system for therapeutic purposes. Indeed, NPs can be functionalized with specific molecules that enable them to selectively bind to immune system cells or to deliver drugs to a specific location in the body [1]. Such advances in nanotechnology have opened new possibilities for targeted drug delivery and precision-medicine applications in the field of cancer treatment and immunotherapy, among others [2,3].

The present review addresses the relationship between the physico-chemical properties of LNPs and their biological effects as they relate to the engagement of the immune system.

## 2. The Immune System

Researchers have clearly shown that LNP surface properties can have an impact on the immune system, which must be considered in addition to the possible effect exerted by the cargo. Avoiding interaction with the immune system is desirable for many therapeutic LNP applications, while specific targeting provides benefits for vaccine delivery and anti-inflammatory, anti-cancer, and anti-viral treatments. Contributions to the immunostimulation effect may come from complement activation, antigen-depot effects, or the possibility that LNPs could transport mRNA to specific cell types or subcellular compartments that favor immune sensing and antigen presentation [3,5]. An extensive description of the complex physiology of the immune system was already well described by Chaplin [6]. Here, the main components of the immune system involved in the recognition and interaction with LNPs will be addressed.

The innate immune response, representing the first line of defense when an unimmunized individual encounters a pathogen, encompasses cell-mediated responses as well as the engagement of the complement system.

The former is dependent on cells from the myeloid lineage. In particular, monocytes, neutrophils, macrophages, and eosinophils are considered true phagocytes, as they can engage, phagocytose, and neutralize their targets, including LNPs. Recognition of these targets, including some lipid-based nanocarriers, is mediated by scavenger receptors (SRs), also called pattern-recognition receptors [7]. SRs, typically expressed by macrophages, mediate the not-opsonic phagocytosis of pathogens or nanoparticles. However, they can also act as co-receptors for the Toll-like receptor (TLR) family, stimulating the release of pro-inflammatory cytokines such as TNFα or IFNγ [8].

The complement system, which involves a complex network of over thirty proteins found in both circulation and cell membranes, plays a crucial role in innate immunity by their recognition and elimination of foreign particles and pathogens by the immune system cells [9].

The complement system favors the elimination of pathogens through opsonization and phagocytosis, while also contributing to chemotaxis and inflammation.

Activation of the complement system involves the deposition of the activated C3b and C3d molecules on pathogens or foreign particles, resulting in increased recognition of antigens by follicular dendritic cells and B cells and induction of the humoral adaptive immune response (described later) [10].

Several soluble factors present in the circulation and in tissues contribute to the fine tuning of complement system activation [3,11]. In fact, dysregulation of the complement system, due for instance to the hypercatabolism of C3 factor, can lead to overt tissue inflammation and toxic effects.

Interestingly, Belling et al. discovered, by working on graphene oxide, that complement activation can be prevented with over 90% efficacy by coating nanoparticles with negative regulatory complement factor H [12]. Similarly, galactose polymer-modified nanoparticles caused lower levels of complement activation compared to glucose-modified nanoparticles, probably due to the adsorption of complement H protein on their surface [13].

On the other hand, some nanocarriers can act as adjuvants and enhance the immunogenicity of weakly antigenic cargo. Indeed, the use of haptenized liposomes, as a research tool to understand the interactions between the complement system and biological membranes, has revealed that all charged phospholipid/cholesterol bilayers can activate complement proteins, although the effects may vary depending on the species, individual, and properties of the vesicles [14].

The fate of nanocarriers upon administration depends on their composition and physico-chemical properties and can result in simple clearance or activation of the immune system also involving the adaptative immunity.

The adaptive immune responses can be classified in cellular immunity, involving the activation of cytotoxic T cells, and humoral immunity, where antigen-specific antibodies are produced by activated B cells. NPs have been reported to activate the adaptative immune response, with factors such as size, charge, hydrophobicity, surface characteristics, solubility, and composition influencing their immunogenicity [15], although the main way to engage immunity is through the release of cargo mRNA expressing specific antigens, such as in the case of LNP-based mRNA vaccines.

## 3. Lipid Nanoparticles

The recent success of the use of LNPs for the COVID-19 vaccine highlighted their importance as delivery systems [16].

The potential of mRNA-induced transient protein expression extends beyond infectious disease vaccines to cancer vaccines, protein replacement therapies, and gene-editing components for rare genetic diseases. In addition to mRNAs, the oligonucleotide development as therapeutics involves silencing RNA (siRNA), antisense oligonucleotides (ASOs), and microRNAs (miRNAs). All of them share relevant delivery challenges, such as sensitivity to degradation by nucleases, renal clearance and sequestration by plasma proteins and the reticuloendothelial system, impaired passage through cell membranes at the intended site of action, and possible lysosomal degradation. Different nanocarriers have been proposed to overcome these limitations; special success was obtained with LNPs, which allowed the commercialization of products such as Patisiran (Onpattro) and recent COVID-19 mRNA vaccines developed by Moderna (Spikevax mRNA-1273^®^) and Pfizer-BioNTech (Comirnaty BNT162b2^®^) [17,18]. According to the literature, the LNP formulation in these products is composed of ionizable lipids, phospholipids, cholesterol, and polyethylene glycol-conjugated lipids (PEG–lipids).

**Figure 2:**
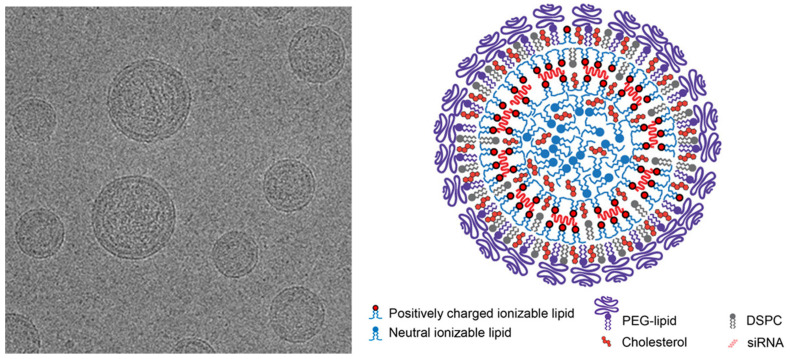
Cryo-TEM image of LNP prepared in the presence of siRNA: LNPs exhibit stacked bilayer structure (**left**); representative image of LNP structure (**right**). Reprinted with permission from [21]. Copyright 2018 American Chemical Society.

LNPs were developed starting from liposomes, lipid-based carriers consisting of a phospholipid bilayer enclosing an aqueous core. However, the next-generation LNPs differ from liposomes in their lipid composition and the internal hydrophobic environment. LNPs are lipid vesicles that have a uniform lipid core and a monolayer lipid shell. According to the literature, it is possible to distinguish liposomes from LNPs, as these are characterized by a multilamellar structure with an electron-dense core [19]. Their internal core is composed of reverse micelles, and they typically range in size from 20 to 200 nm. Moreover, compared to liposomes, they show greater physical stability and a more complex internal structure, which is still under investigation [20] (Figure 2).

Among the researchers that deeply investigated the structural dynamics of LNPs and their interaction with RNA molecules, Kulkarni et al. studied, by cryo-TEM and X-ray, empty and siRNA-loaded LNP formations before and after changing the pH from 4.0 to the physiological pH 7.4. Empty LNPs exhibited at pH 4.0 a lamellar structure, while an electron-dense amorphous solid core occurred at pH 7.4, increasing with the increasing amount of the ionizable lipid used (DLin-KC2-DMA); this electron-dense structure was not evidenced in the case of LNPs based on a permanently cationic lipid (DOTMA). High siRNA contents (nitrogen to phosphorus ratio—N/P ~ 1) resulted in concentric bilayer ring structures, while slightly elevated N/P ratios (1.1–1.5) showed concentric rings and an amorphous core. A further increase in the N/P ratio to the most common values of 3 and 6 resulted in siRNA–lipid bilayers at the outer portions of LNPs, with amorphous lipids at the structure core [21]. Another study, based on the application of dynamic nuclear polarization (DNP) NMR spectroscopy on Lin-MC3-DMA LNPs loaded with siRNA or mRNA, proposes an internal structure with a core made up of inverted micelles and cholesterol, covered with a phospholipid monolayer, that encompasses the nucleic acid as the active principle [22]. A quite recent study on Comirnaty^®^ formulation, using TEM, cryo-TEM, atomic force microscopy (AFM), and force spectroscopy, suggests the presence in the lipid core of labyrinth-like networks of lipid-RNA assemblies stabilized by hydrogen bonding, considering the lack of ionization of the lipid [23]. These findings propose a refined model for the LNP-RNA structure, shedding light on the intricate interplay crucial for therapeutic formulations. As recently pointed out by Albertsen, it is likely that even small changes not only in the main lipid but also in other components, such as cholesterol, can induce changes in the LNP structure, able in turn to impact biological aspects, pharmacokinetics, interaction with cells, and immunological effects [24].

The presence of an ionizable lipid in the LNP formulation seems to influence the LNP cellular uptake; In fact, it is partially positively charged at a pH value below its acid-dissociation constant (pKa) and interacts with a negatively charged membrane [24]. Moreover, it has been proposed that as the endosomal pH decreases, due to the fusion between lysosomes and early endosomes, the LNP surface becomes positively charged and interacts with the negatively charged endosomal membrane. This mechanism brings the disruption of the endosomal membrane and the subsequent release of the cargo [21].

COVID-19 mRNA vaccines administered in the deltoid muscle in humans stimulate inflammation and recruitment of neutrophils, monocytes, and dendritic cells. In a mouse model, it has been observed that the subcutaneous administration route of the COVID-19 vaccine Comirnaty^®^ gave a lower pro-inflammatory response with no reduction in vaccine efficacy. Better knowledge about the role of ionizable lipids and of LNP physicochemical properties for the uptake and transfection in the different immune cells, like dendritic cells, could be useful for further vaccine optimization and for the use of LNPs in chronic infections, gene therapy, and cancer treatment [25].

Qin et al. showed that LNP-based vaccines cause long-term changes in the immune system, mostly affecting the adaptive immune response and, subsequently, the protection against infections [26]. Enhanced insight into the origins of reactogenicity could pave the way for the development of advanced mRNA-LNP vaccines; these could potentially elicit more robust and enduring adaptive immune responses, all while reducing the occurrence of moderate and severe adverse reactions, including rare instances of anaphylaxis and acute myocarditis/pericarditis, in vaccinated individuals [5].

Different recent studies, also performed with empty LNPs, demonstrate their ability to perform as vaccine adjuvants beyond the presence of mRNA cargo. In Swaminathan et al., for example, LNPs were either co-administered or used as vehicles of a TLR9 agonist, showing a response in antibody production higher than that obtained with TLR9 alone. The positive response was also observed for LNPs alone, which induced a Th1-type immune response [27]. The production of pro-inflammatory cytokine IL-6 was observed after LNP administration to mice, triggering the differentiation of T cells to Th cells, and it was identified as critical for the adjuvant activity of LNPs [28]. However, the contribution of LNP carriers to immunostimulation in supporting vaccine efficacy must be balanced with the immunotoxicity that can arise from excess stimulation. All these considerations encourage the use of unloaded LNPs as the control group in the study of LNP-RNA products [29].

The physico-chemical characteristics of LNPs, including size, shape, elasticity, surface chemistry, and composition, play a crucial role in influencing LNPs’ biodistribution, RNA delivery, and interaction with immune system cells [30]. Techniques used to examine these attributes can be adjusted to optimize LNP formulations. Looking at the future of RNA-based therapies, there are still questions to be answered in LNP formulation and optimization [30,31].

## 4. Effect of Lipid Nanoparticle Physico-Chemical Properties on Cell Internalization and Interaction with the Immune System

**Figure 3:**
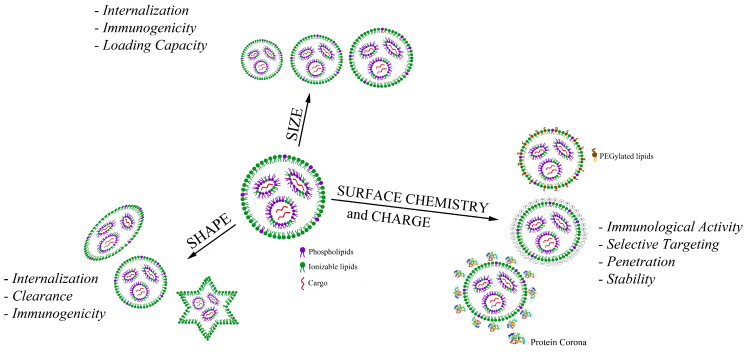
Schematic illustration of biological properties affected by the LNP physico-chemical properties.

This section presents the effects of different physico-chemical properties of LNPs on their cell internalization and interaction with the immune system. In particular, we considered particle size, in terms of size distribution and polydispersity index, shape, elasticity, surface chemistry, and surface charge as the main LNP characteristics that influence the particle fate after the administration (Figure 3).

### 4.1. Size

The quality and suitability of LNPs for various applications depend on their mean size and size distribution. The dimensions and polydispersion index, indicating dimensional distribution, are often determined by dynamic light scattering (DLS), which remains the most diffused technique, although more and more clear is the usefulness of orthogonal characterization also involving different analytical methods, among which are separation methods such as SEC (size exclusion chromatography) and FFF (field flow fractionation) and methods involving single particle detection such as nanoparticle tracking analysis (NTA) and tunable resistive pulse sensing (TRPS) [32,33].

As described in detail by Rennick et al. [34], the endocytosis involving clathrin and dynamin-dependent mechanism (CME), which is present in all mammalian cells, is activated for spherical nanoparticles with a size lower than 100 nm, corresponding to the size of clathrin-coated vesicles, although larger non-spherical NPs can be internalized through actin involvement. Also considering fast endophilin-mediated endocytosis (FEME) and glycosylphosphatidylinositol-anchored protein-enriched early endocytic compartment (GEEC) endocytosis, it can be considered that the upper limit of NPs’ size for internalization is about 200 nm. For particles with dimensions higher than 200 nm, internalization relies on other mechanisms such as micropinocytosis or phagocytosis [34].

The size of NPs also plays a crucial role in different biological phenomena such as circulation half-lives, vascular extravasation, macrophage uptake, and in vivo distribution. NPs with a very low mean diameter, below ~5 nm, undergo rapid renal clearance, while those exceeding 200 nm face splenic filtration. NPs around 100 nm exhibit prolonged circulation, enhancing extravasation by the EPR effect, which is sensitive to tumor vasculature fenestrations. These generally vary in the range from 380 to 780 nm, but in less permeable tumors like the pancreatic adenocarcinoma, fenestration limits the passage to NPs of less than 50 nm [35]. In the liver, the fenestra diameter of about 100 nm limits the penetration of low-dimension nanoparticles, although liver sinusoidal endothelial cells (LSECs) show higher endocytosis, so that they can be targeted by larger particles.

Ji and colleagues developed and compared three different lipid-based particle formulations, loaded with mRNA and characterized by three mean diameters: 90 nm (low LNPs), 300 nm (medium LNPs), and 1150 nm (micro LPs) [36]. Experimental results demonstrated that low LNPs and micro LPs showed higher in vitro transfection efficacy with respect to medium LNPs. In addition, it was demonstrated that low LNPs and micro LPs showed a similar robust immune response and excellent intracellular delivery ability.

In another study, LNPs loaded with siRNA and subcutaneously administrated resulted in different silencing FVII effects: in detail, LNPs with the lowest (30 nm) and highest (80 nm) dimensions presented lower silencing ability with respect to the intermediate-size LNPs (45 nm). The accumulation in the liver was, however, inversely proportional to the dimensions and, therefore, highest for 30 nm LNPs. These results can be explained by hypothesizing the better delivery of small LNPs to regional lymph nodes. Moreover, it is suggested that 30 nm LNPs can more easily penetrate tumors even if characterized by poor permeability [37].

In light of these considerations, the optimal size range for LNPs is considered to be approximately 20–200 nm, as this size enables them to withstand fluid flow (e.g., blood and lymph) while crossing the interstitium and larger particles (>200 nm) accumulate in the liver, spleen, and lung cells, although distribution is influenced by a combination of size, shape, and surface charge effects [35,38].

In the literature, many interesting works analyzed the effects of non-lipidic NPs on their interactions with the immune system; unfortunately, not many researchers have studied this aspect with a specific focus on LNPs. Here, we reported the main interesting data that we think could be applied to LNPs.

A good survey of the literature concerning the relationship between physico-chemical properties of NPs and their interaction with the immune system was created by Dobrovolskaia et al. [3]. In general, NPs with a mean size lower than 100 nm and, even better, around 50 nm, are better internalized by dendritic cells, while larger NPs are better internalized by macrophages [3]. Particles with a size distribution of about 20–200 nm generally elicit stronger immune responses than larger ones. Smaller particles utilize both cell-associated migration and lymphatic drainage, providing better antigen presentation. Particle size has also been suggested as a primary factor in determining the type of immunity induced, with larger particles (>1 µm) generally associated with inducing a Th1 response and smaller nanoparticles (<500 nm) more specifically inducing the Th2 responses [3,39]. Specific studies concerning complement activation have shown that, for polystyrene NPs functionalized with IgG, larger particles with diameters exceeding a few microns demonstrate significantly reduced complement activation [40]. Further studies put in evidence that the M1 polarization relationship was seen with graphene oxide NP dimensions, and polystyrene NPs of different dimensions showed selective interaction with immune cells, as they were taken up by dendritic cells preferentially at the site of injection when the dimensions were large (over 500 nm) and in lymph node DC and macrophages when the dimensions were small (20–200 nm). For polystyrene NPs, both the humoral response has resulted in being higher for small dimensions (<100 nm), and in general, a Th2 response has been observed with NPs lower than 500 nm, with only some exceptions observed, among which are PLGA NPs and nanoemulsions that induced Th1 response in case of smaller dimensions [14].

The effect of the NP size on the direction of the immune response toward Th1 or Th2 phenotype was seen in the case of lipid vesicles in an early study by Brewer et al. (1998), who observed that endocytosis by macrophages of vesicles with a diameter higher than 225 nm induced IL-12 production and prevalent Th1 response, while exposure to vesicles lower than 155 nm resulted in the prevalence of Th2 through IL-1β production [41]. The impact of vesicle size on Th1/Th2 response was confirmed by Mann et al. for liposomes containing bile salts (bilosomes) loaded with influenza A antigen and aimed at oral mucosal immunization. The batch with two populations of larger particle size (mean diameter 980 nm), administered to BALB/c mice, resulted in a prevalent Th1 response and greater response than the batch of small dimensions (mean diameter 250 nm) [42]. This effect could be related to the different “drug” loading of small and big liposomes: vesicles with higher mean diameter can load a higher dose of cargo which could be directly correlated with the Th1 responses [43].

In their study about adjuvant properties of LNPs, Swaminathan et al. considered samples of approximately 100 nm in diameter, but they also reported that different sizes (60, 160, and 250 nm) did not significantly impact adjuvant properties [27].

More recently, a specific study about the impact of NP dimensions on immunogenic response was performed by Hasset et al. by assessing mRNA-LNPs vaccine encoding for a cytomegalovirus surface pentamer complex and for envelope glycoprotein gB. In a murine model, they observed, for pentamer antibodies, a clear increase in the titer with the dimensions up to about 100 nm for the first dose and up to 85 nm for the second dose. In the case of glycoprotein gB titer, a clear increase with the increase in dimensions was observed only for the second dose, with LNPs up to 110 nm. Above these dimensions, a plateau of response was obtained, so that for particles exceeding about 100 nm in size and up to 200 nm, antibody titers consistently remain elevated. Therefore, in mice it has been seen that LNPs below 85–100 nm induced significantly lower immunogenicity with respect to the size interval of 100–200 nm. According to the authors, all the tested samples had a size favorable for phagocytosis, but were possibly subject to different kinetics of cell entry. Conversely, all sizes of particles that were investigated triggered a strong immune response in non-human primates which appeared to be less sensitive to particle size when LNPs are delivered intramuscularly, as compared to rodents. This result can be explained by higher dimensions of lymphatic vessels in primates, which did not limit the drainage of LNPs within the size range studied [44,45].

The size of LNPs can be modulated by modulating the preparative mixing parameters, such as, for a microfluidic process, flow rate, and volume ratio, and by altering the cholesterol, lipids, and PEG–lipid structure and percentage. In particular, PEG–lipids prevent aggregation and lead to the formation of a homogeneous population of particles [46].

However, it must be remembered that as soon as NPs enter in contact with biological fluids, a protein corona formation occurs that can change the dimensions with respect to those measured after preparation. Another effect that must be considered concerns particle aggregation before reaching the target cells [34]. In our opinion, all NP formulation should be characterized in terms of size distribution not only in static conditions but also after their interaction with plasma or blood to better simulate its behavior after in vivo administration.

### 4.2. Shape and Elasticity

The shape of NPs is a crucial determinant of cellular uptake, according to the literature, possibly more than size. Research has shown that NPs with a wrinkled surface are characterized by increased cellular uptake because the roughness promotes the interaction of the particle with the cell membrane, through the formation of nonspecific binding forces [47].

Elongated particles are more efficient at delivering drugs, as they avoid internalization into phagocytic cells, as demonstrated for polystyrene beads with a disk shape, that have longer half-lives in circulation and more specificity of targeting in mice models compared to their spherical counterparts [48]. Worm-shaped PEG micelles have also exhibited prolonged circulation time and tumor accumulation in rodents [14,49]. However, the higher contact area of elongated nanosystems with cells may lead to more damage to the cell membrane. Wibroe and colleagues studied the carboxylated polystyrene NPs of dimensions lower than 500 nm and of different shapes, compared at the same surface area, and evaluated the complement activation in human and pig blood and in the pig model. In pig blood, complement activation occurred only after 5 min of contact, and was more intense for rods and disks than for spheres. In human blood, delayed complement activation was instead similar for the different shapes. A possible role of different protein corona adsorption was envisaged for this different behavior. In a pig model, rods and disks did not induce any notable cardiopulmonary distress when compared to spheres. The authors showed that spherical particles were more rapidly cleared from the blood as compared to non-spherical rods and disks, for which the slower clearance rate seemed related to a desensitization process and to a lower cardiopulmonary negative reaction. Immediate and robust particle phagocytosis by resident pulmonary intravascular macrophages, faster for spheres, may lead to cardiopulmonary responses [50].

Specifically, deepening the effect of shape in LNPs, Cao et al. studied star-shaped lipid nanocarriers based on phosphatidylcholine with different tail lengths. They found that lipid nanostars demonstrated increased cellular uptake in human liver carcinoma cells (HepG2) and in triple-negative breast cancer cells (4T1) and an enhanced tumor extravasation in vivo compared to spherical LNPs. In terms of the immunogenicity of lipid nanostars, this was studied in Balb/c mice after 24 h from the intravenous injection, and by analyzing serum aspartate transaminase, alanine transaminase, urea, and creatinine, no significant changes were reported compared to spherical LNPs [51].

It was also discovered that cholesterol-modified LNPs take on a polyhedral form, not spherical, displaying multilamellarity and lipid partitioning. Those with cholesteryl oleate exhibit greater affinity for liver endothelial cells than hepatocytes. Oxidative changes on the cholesterol tail enhance accumulation in liver endothelial cells and Kupffer cells [52].

Researchers have also studied the shape and structure modifications occurring by using phytosterols as cholesterol analogs in LNP formulation. The authors found that by using beta-sitosterol as the cholesterol phase, the LNP shape could be changed from being regularly spherical to highly faced, corresponding to an increase in fusion with the HeLa cell membrane, and higher internalization. More efficient transfection, up to ten times that obtained with cholesterol LNPs, was also observed in this case, explained by easier fusion with endosome membranes and higher RNA endosomal escape [53]. Other phytosterols, such as fucosterol, campesterol, and stigmastanol, changed the inner LNP structure inducing multilamellarity and lipid partitioning [54].

NPs’ conformation can influence their immunogenicity. Spherical particles are less prone to activate the complement system compared to rod- and disk-shaped NPs. It has also been observed that the deposition of complement protein fragments on the NP surface, such as liposomes, can significantly affect their blood-level kinetics and particle integrity [55].

Elasticity is another feature that was poorly investigated on LNPs. Li et al., in studying polymeric NPs, reported that their pliability has a significant impact on their biomedical effects, including pharmacokinetics and in vivo targeting, which can influence clearance mechanisms and off-target behavior. Specifically, flexible NPs have slower clearance rates compared to stiff ones. For example, red blood cells offer a natural model for designing materials that can circulate in the blood for extended periods without causing harm, as they can evade splenic filtration and pass through capillaries with diameters one-tenth of their size [56]. Highly flexible hydrogel-based NPs, designed to mimic red blood cells, have a circulation time that is longer compared to similar particles with higher crosslinker density [57].

The flexibility of NPs can also affect particle interaction with immune cells, in particular with macrophages. In vivo studies have shown that soft polymeric NPs are removed by the mononuclear phagocyte system to a lesser extent than more rigid ones [58].

Baranov et al. used a computational approach to predict the interaction of NPs with different sizes and shapes with cell membranes and performed a comparison with available experimental data. Regarding the shape, computational methods resulted partially in line with experimental data suggesting an easier cell uptake for spherical than for ellipsoid NPs. For rigid NPs, better uptake is predicted, explained by the higher energy and the involvement of more receptors that the presence of particle deformation requires for membrane wrapping. In line with that, rigid particles are consumed more efficiently by mouse bone marrow-derived macrophages and macrophage-like cell lines than softer ones, as soft particles are more easily deformed during the phagocytic process [59].

In recent investigations, Li et al. found a relationship between NP elasticity and ApoA1 adsorption, observing that ApoA1 displays a notable affinity for NPs of intermediate elasticity, with values in the range of 75–700 kPa. Assays conducted to study the effect of ApoA1 liposomes, on both suspended and adherent macrophages to better simulate the in vivo conditions, demonstrated that they suppress cellular uptake, reducing cytotoxicity and inflammation [60].

Following these findings, it was suggested that immunization with vaccines based on more rigid lipid vesicles can result in higher antibody expression and T-cell response [61].

### 4.3. Surface Chemistry and Charge

The surface chemistry and charge of LNPs play a critical role in their interactions with cellular membranes and the biological environment, including immunological activity. In particular, the surface charge of LNPs determines their uptake by cells: if LNPs have a negative surface charge, they undergo limited interaction with the negatively charged cell membrane and are hardly taken up by cells, while conversely, positively charged LNPs can disrupt cellular membranes and cause toxicity. Highly positively charged nucleic acid carriers can effectively interact with cell membranes, leading to lysosomal rupture, high cargo release, and improved transfection efficiency [62]. However, this same effective interaction may also lead to membrane disruption and toxicity, especially when high doses are used [20].

To address this issue, ionizable lipids are incorporated into LNP designs aimed at loading with siRNA like Onpattro^®^, or with mRNA such as with COVID-19 vaccines. The carefully designed logKa of base lipids, as explained hereafter, makes a possible ionic interaction with RNA at low pH values during RNA loading but leads to non-ionic NPs at the moment of administration and cell interaction; but the positive charge is again acquired at acidic endosomal pH. The surface charge of LNPs is commonly measured using zeta potential and can be modulated by choosing the lipid pKa, and, for RNA-loaded LNPs, by adjusting the N/P ratio, that is the ratio between ionizable lipids and nucleic acid. Increasing the N/P ratio has been shown to increase surface charge and encapsulation efficiency, but protonation levels at the endosomal pH can decrease, decreasing LNP potency. Choice of lipid and N/P ratio also affected LNP trafficking after IV or IM administration, with, for example, higher off-target liver expression after IM for more negative LNPs [63].

It is interesting to consider how permanently charged lipids can be incorporated into LNPs to achieve selective organ targeting. For example, positively charged lipids were added to LNP formulations to preferentially transfect lung tissues, while negatively charged lipids directed transfection toward the spleen [64].

Regarding charge-related toxicity, Collodel et al. found that both anionic and cationic liposomes induced apoptosis in neonatal porcine Sertoli cells, while zwitterionic liposomes were better tolerated [65].

Charged NPs, especially cationic ones, are more readily phagocytosed and more quickly cleared by macrophages, and can trigger the production of pro-inflammatory cytokines [66]. This is the rationale of the use of cationic liposomes as vaccine adjuvants for poorly immunogenic antigens [67,68,69]. Especially useful for this purpose are the cationic lipids with unsaturated tails or short saturated tails, for their higher immunomodulatory activity [29,70]. Cationic lipids can activate TLRs, leading to the induction of pro-inflammatory cytokines and other molecules that co-stimulate the immune system [71,72].

A panel of cationic lipids, among which are dimethyl-dioctadecyl ammonium (DDA) and 1,2-di-(9Z-octadecenoyl)-3-trimethylammonium-propane (DOTAP), have been compared with MC3 as benchmark ionizable lipids in LNPs loaded with RNA encoding rabies virus protein (RGV). They were studied in vitro in baby hamster kidney cells (BHK) by evaluating antigen expression which resulted in being higher in DDA and DOTAP LNPs than for control MC3 LNPs. The immunogenic response in BALB/c mice by IM injection was instead higher for MC3 at low concentrations and comparable at high dosages [73].

Cationic lipids, such as DDA, have been shown to increase the immunogenicity of LNP-formulated mRNA vaccines [67]. DDA can potentially enhance vaccine efficacy, increasing both cell-mediated and humoral immune responses [74]. This characteristic derives from their ability to interact with the innate immune system, specifically by inducing ionic interactions with antigens (Ags) [68,75], promoting uptake by antigen-presenting cells (APCs) and improving expression of maturation markers in APCs [75,76]. Additionally, it has been demonstrated that LNPs containing DOTAP and 1,2-di-O-octadecenyl-3-trimethylammonium propane (DOTMA) can activate TLRs and NLRP3 inflammasome pathways [31].

Blakney et al. considered both cationic and ionizable lipids as carriers of self-amplifying RNA (saRNA) as anti-infective and anti-cancer vaccination. They observed that the cationic LNPs based on DDA and DOTAP were able to protect RNA from enzymatic degradation even when it was adsorbed at the LNP surface. Delivery efficiency and antibody production in BALB/c mice were comparable for both cationic and ionizable LNPs, prepared using C12-200 lipid [75].

However, cationic particles are cytotoxic to a range of immune cells, induce TLR and proinflammatory cytokine secretion [29,77], exaggerate endotoxin-mediated toxicities, activate complement, bind plasma proteins, trigger pro-coagulant activity, and potentially affect protein conformation and function [14,66,78,79]. This is especially true for quaternary ammonium lipids, while headgroups that allow charge delocalization, such as imidazolium, guanidinium, and pyridinium, resulted in lower toxicity effects [80].

Anionic NPs can also cause adverse effects, in some cases induced by the adsorption of proteins such as fibrinogen, inducing inflammatory cytokine release via the integrin receptor, Mac-1 [81]. Negative surfaces are reported to interact with scavenger receptors involved in the activation of the immune system and induction of pro-inflammatory cytokine release [14,81,82]. Negatively charged NPs primarily activate the classical complement cascade, while their positively charged counterparts more commonly induce the alternative pathway, involving opsonization by plasma proteins. This phenomenon is known to be sensitive to charge, surface coatings, and their thickness and density, and accessibility to reactive groups. The current more diffuse strategy to limit protein adsorption on the NP surface relies on PEG coating. However, further studies seem necessary to modulate complement activation through the modification of the NP surface, to further improve its protection towards the protein corona [83].

Despite the advantages related to the use of cationic lipids, until now the commercially available RNA-loaded LNPs contain two ionizable lipids: heptadecan-9-yl 8-[(2-hydroxyethyl)[6-oxo-6-(undecyloxy)hexyl]amino]octanoate (SM-102) and a mixture of ALC-0315 and ALC-0159 ((4-hydroxybutyl)azanediyl)bis(hexane-6,1-diyl)bis(2-hexyldecanoate) and 2-[(polyethylene glycol)-2000]-N,N-ditetradecylacetamide) for Spikevax^®^ and Comirnaty^®^, respectively.

Thanks to chemical research, much progress has been made and, in particular, Gueguen and colleagues synthesized a new permanent positively charged lipid that presents a low toxicity profile, similar to the ionizable lipids used for the commercially available LNPs, but higher cell internalization and nucleic acid delivery. In detail, the quaternary ammonium group was substituted with a new H-heterocycle, imidazolium, which carried the positive charge [84].

The advantages and limits related to the use of cationic and ionizable lipids are reported in Table 1.

The LNP surface could also be modified by binding targeting ligands, like antibodies, as a valid strategy for specifically targeting drug delivery, but this can increase their immunogenicity and impact pharmacokinetics [14].

Table 2 summarizes the LNP physico-chemical properties, the principal techniques used for their determination, and their biological in vitro/in vivo effects.

## 5. Effect of Lipid Nanoparticle Materials

**Figure 4:**
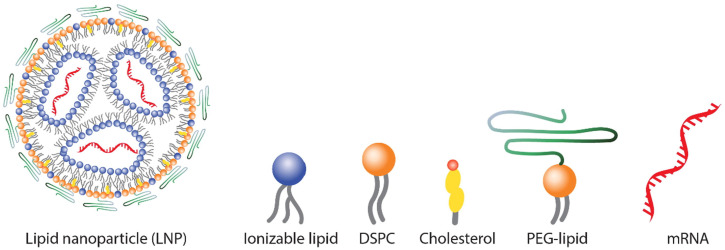
Illustration of LNPs structure and components. Reprinted with permission [24].

All of the currently FDA-approved LNP formulations contain four lipids: an ionizable cationic lipid, responsible for RNA loading and release behavior, two helper lipids useful to stabilize the LNPs (which include 1,2-distearoyl-sn-glycero-3-phosphocholine (DSPC) and cholesterol), and a PEG–lipid conjugate able to modulate the LNP surface properties [20] (Figure 4). These constituents promote the formation of a monodisperse LNP population, promote LNP stability, enable efficient encapsulation of nucleic acid, enhance cellular uptake, and promote endosomal escape of nucleic acid cargo. Lipid nanocarriers have been shown to contribute to the drug’s immunostimulation and these safety data have raised concern [87,88]. Immunostimulatory lipids, such as 3-dimethylamino-2-(Cholest-5-en-3βoxybutan-4-oxy)-1-(cis,cis-9,12-octadecadienoxy)propane (C-Lin-DMA), PEG-dimyristoylglycerol, protamine, trimethyl ammonium propane-cholesterol, and DOTAP, are commonly used to prepare nanocarriers for therapeutic nucleic acid delivery [87,89,90,91]. Clinical studies investigating the safety of such formulations often involve premedicating patients with immunosuppressive cocktails containing immunosuppressive agents, antipyretic agents, and histamine H1 and H2 receptor blockers [92].

In this chapter, we will consider each LNP main component and its effect on different biological mechanisms, such as cellular uptake, cell interaction, immunostimulation, and RNA delivery efficiency.

### 5.1. Ionizable Cationic Lipids

**Figure 5:**
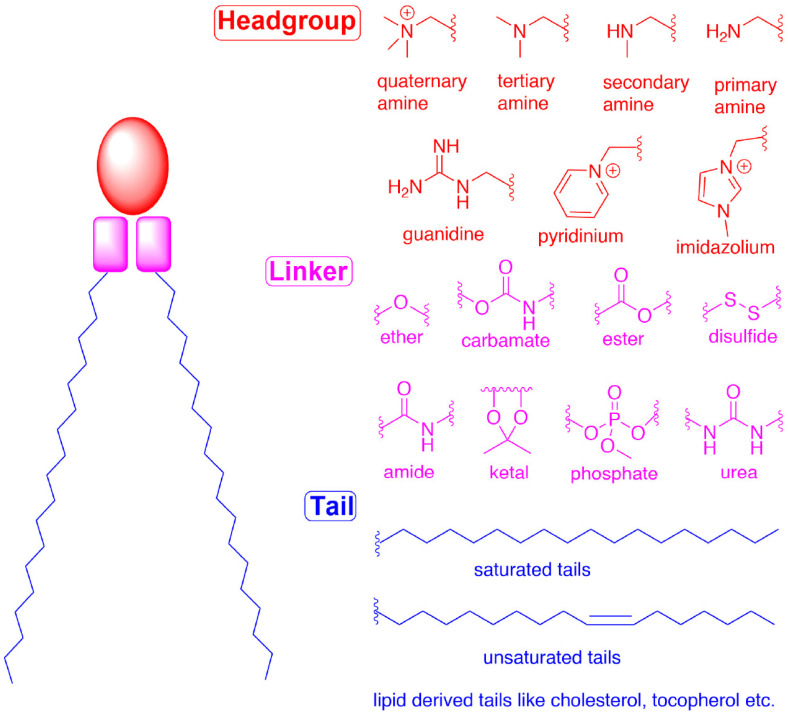
Schematic representation of cationic and ionizable lipids and their components (headgroup, linker, and tail). Reprinted with permission from [30]. Copyright 2022 American Chemical Society.

During the RNA-loaded LNP formation process, positively charged ionizable lipids facilitate the integration of nucleic acids into the emerging NP through electrostatic interactions with the negatively charged phosphate backbone of nucleic acid polymers. This mechanism allowed the formation of inversed micelles into the core of LNPs which encapsulate and protect nucleic acids into the nanostructures (Figure 5). To guarantee effective ionizable lipid protonation, LNPs are assembled at a pH value below the pKa value of ionizable lipids which is usually around 6.5, followed by an exchanging step of buffers at physiological pH values [24] (Figure 4).

The use of ionizable cationic lipids helps to avoid the harmful cytotoxic effects that are often associated with permanently charged cationic lipids. Their peculiarity lies in the acquisition of a transient cationic charge at the low pH values, used during LNP production, resulting in the formation of reverse micelles that encapsulate oligonucleotides within the LNP core, at typically more than 90% efficiency [93]. A positive charge occurs again when LNPs reach the endosomal compartment, leading to destabilization of the endosomal membrane and RNA release in the cytosol [85]. At pH 7.4, the LNPs are close to neutrality, resulting in lower toxicity and prolonged plasma half-life for lower interaction with plasma proteins.

Among the pioneer works about ionizable lipids for siRNA delivery, Zhang et al., studying ionization effects of LNP components, put in evidence the pKa value of amino–lipids as a key feature for NP behavior. They compared pKa prediction with different experimental methods to determine amino–lipid pKa, such as potentiometric titration and TNS fluorescence. A relationship between pKa and cell membrane interaction was also studied using carboxyfluorescein as a reporter for lysis, and put in evidence that the interaction between lipid and membrane is highest for the highest amino–lipid charge [94].

In the last decade, efforts in optimizing ionizable lipids led to a vast choice of different chemical structures, whose properties have been recently extensively reviewed [29,95]. Quite recently, Naidu et al., using both in vitro epithelial, fibroblast, and macrophage cell lines and an in vivo mice model, demonstrated that the selection of different combinations of linkers and hydrophobic tails could improve the cell-type specificity of mRNA delivery [96]. The ionizable lipid structure is more and more clearly recognized as responsible for functionality, toxicity, and immunostimulation. Linker biodegradability improves clearance and reduces toxicity, while immunoactivity seems especially related to head structure, as it determines the pKa of the lipid. Quaternary ammonium moieties appear more toxic than tertiary ones for interaction with protein kinase C. As already pointed out, unloaded LNPs demonstrated immunostimulation and adjuvant activity in vaccine activity [29,30,80].

The ionizable lipids commercially used in COVID-19 vaccines, such as Comirnaty^®^, Spikevax^®^, and Onpattro^®^, are subject to hydrolysis degradation as they contain esters. Although DLin-MC3-DMA (MC3—heptatriaconta-6,9,28,31-tetraen-19-yl-4-(dimethylamino)butanoate) lipid, used in Onpattro^®^ formulation, is not completely biodegradable, clinical doses show no signs of toxicity [97].

Hassett et al. tested various LNPs formulations in mice, looking for an ionizable lipid with better performance than MC3, previously used in Onpattro^®^ formulation. The tested ionizable lipids were based on tertiary amine and were compared with a quaternary one (N-[1-(2,3-Dioleoyloxy)propyl]-N,N,N trimethylammonium—DOTAP). DOTAP did not show protein expression or immunogenicity. The authors found that mRNA-LNP immunogenicity was significantly influenced by the ionizable lipid pKa with the best immunogenicity corresponding to a pH range of 6.6–6.9, slightly surpassing the typical optimal pKa (6.2–6.5) for IV administration to the liver. This suggests that lipid pKa might impact interactions between the immune system and the formulation, although other factors can play a role. Moreover, it must be considered that endosomal acidification can be different according to the tissue and the cell line. It was also found that a faster biodegradability reduced inflammation at the site of injection. Among the different structures analyzed, the one with pKa 6.68 and optimized biodegradability was selected as the lead compound [98]. The more recent work of Carrasco et al. compared the pKa parameter obtained by different theoretical and experimental methods such as TNS binding, NMR, and Z potential titration for five known ionizable lipids: DLin-KC2-DMA, DLin-MC3-DMA, DLin-DMA, DODMA, and DODAP. The low pKa (5.62) of DODAP, too low for endosomal escape, explains its poor transfection in HEK293 cells. KC2 resulted in higher transfection than MC3, explained by its less negative charge leading to better interaction with negative cell membranes. The same order was found in mice after IM injection, which involves the presence of negative proteoglycans, while after IV administration, MC3-based LNPs sustained higher expression in the liver. A possible role of APO-E adsorption for negative LNPs is envisaged [63].

The use of an imidazole structure as a head group for the ionizable lipid was suggested by Ripoli et al., taking into account its suitable pKa, in the range of 5.5–6.5, obtaining a strong immune response in mice and monkeys, besides increased LNP stability [99]. The buffering effect of the imidazole ring was also exploited by Liu et al., who synthesized imidazole derivatives with different linkers paying attention to lipid pKa, whose value was set at 6.39 for the optimal endosomal escape of siRNA-loaded LNPs [100]. The relevance of pKa was not so evident from the work of Bernard et al. who studied the relevance of the ionizable lipid structure on the immunogenicity of a modified mRNA vaccine. They compared LNPs differing for the ionizable lipid that were MC3 (pKa 6.4), and two modifications that maintained the same head group of MC3 but differed for the lipid tail that presented hydrolyzable ester bonds for L319 (pKa 6.4) or the presence of an ester linker for KC2 (pKa 6.7). The dimensions of the LNPs were higher (160 nm) for L319 than for MC3 and KC2 (80 nm), although in all cases, they were small enough to allow lymph node drainage. Reporter luciferase expression was higher with L319 and MC3 LNPs at the site of injection and lymph nodes but also in the liver and spleen. In this study, luciferase transfection was in line with the immune response in mice, with a higher response for L319. The inverse correlation between immune response and increase in IFN-α titer, which was higher for KC2 and MC3 LNPs, was confirmed. RNA modification impacted innate chemokine and cytokine induction and antibody titers to a lower extent for L319 than for MC3 and KC2 [101].

### 5.2. Helper Lipids

LNP formulation comprises auxiliary lipids, including cholesterol, a phospholipid, and a lipid with PEGylation. They are known as helper lipids as their primary role is to support the structure, ensure colloidal stability, contribute to gaining high encapsulation efficiency, and regulate the size of the LNPs [102]. Helper lipids play a crucial role in maintaining the stability of the LNPs during their production, storage, and circulation. This term encompasses a variety of lipids, including phospholipids, sterols, and glycerolipids, which are typically not cationic. However, surfactant and PEG–lipids have also been referred to as helper lipids [24].

#### 5.2.1. Cholesterol

Cholesterol plays a role in enhancing particle stability by increasing membrane integrity and rigidity and contributing to the shift of the lipid structure to a liquid-ordered phase. Additionally, the choice of cholesterol derivatives affects the shape of the LNPs and their multilamellar structure [24,53]. Among the structural characteristics that influence the effectiveness of cholesterol analogs for in vivo delivery, there are the flexibility of sterol rings, the polarity of hydroxyl groups, and in particular, the length of the hydrophobic alkyl chain. In particular, Patel et al., by studying different sterols in LNPs, found that the body region of the cholesterol rings must be maintained to preserve transfection, as demonstrated by the lack of efficacy obtained with vitamin D2 and D3. Good transfection improvement was obtained in the presence of a C24 ethyl group like in β-Sitosterol, probably for better interaction with cell membranes [53].

In a further study, the same authors modified cholesterol with hydroxy substitutions, either in the body or in the tail positions. They evaluated the developed LNP formulations, containing the proposed cholesterol derivatives, for mRNA delivery to primary human T cells. They found that increasing the percentage of 7α hydroxy substitution from 25% to 50% enhanced mRNA delivery from 1.8-fold to 2.0-fold, suggesting a promising application to immunotherapy. This can be attributed to the relationship between the increase in substitution and the increase in pKa, so that LNPs can release mRNA at the higher pH values of early endosomal trafficking [103].

The synthesis of ionizable derivatives of cholesterol conjugated with histidinamide brought the improvement of the cytoplasmatic delivery of LNPs by modulating the pKa in the pH 6–7 range, optimal for endosomal escape [104].

The molecular structure of cholesterol derivatives can also impact the delivery effectiveness and biodistribution of LNPs, reducing the protein adsorption surface and increasing circulation time [24]. Cholesteryl oleate exhibits increased selectivity for liver endothelial cells over hepatocytes. Furthermore, oxidative modifications on the cholesterol tail enable LNPs to accumulate more in liver endothelial cells and Kupffer cells compared to hepatocytes [52].

#### 5.2.2. Phospholipids

Among helper lipids, phospholipids play an important role helping in the packaging of nucleic acids and stabilizing LNPs during storage. Phospholipids were the major components (about 85 mol%) of the original lipid-based nanoparticles from which the LNP is derived. Subsequently, their concentration has decreased in favor of the ionizable lipids, and in the current commercial LNP formulations, phospholipids typically constitute about 10–20% of the total lipid amount. Phospholipids are used as structural components due to their ability to spontaneously organize into lipid bilayers with high phase transition temperatures that confer the LNP membrane stability. They also aid the encapsulation efficiency and endosomal escape [105].

A typical phospholipid is an amphiphilic molecule, composed of glycerol, two hydrophobic fatty acid tails, and a phosphate-linked head group. Both semisynthetic, generally derived from phosphatidylcholine phospholipids and natural phospholipids can be used to formulate LNPs for RNA delivery where they are located at the periphery of the structure [80]. However, the use of semisynthetic lipids is preferred because of their purity, commercial availability, chemical functionality, and cost effectiveness, and those most commonly utilized include phosphatidylcholines, phosphatidylethanolamines, and phosphatidylglycerols, such as 1,2-distearoyl-snglycero-3-phosphocholine (DSPC) and 1,2-dioleoyl-snglycero-3-phosphocholine (DOPC), 1,2-dioleoyl-sn-glycero-3-phosphoethanolamine (DOPE), and dioleoyl phosphatidylglycerol (DOPG) [106].

The DSPC is a structural phosphatidylcholine-derived lipid that is used in the first clinically approved LNP formulation, such as a siRNA therapeutic Onpattro^®^ (patisiran) and mRNA vaccines against SARS-CoV-2 [20,107]. Structurally, DSPC is composed of a phosphatidylcholine headgroup and two saturated C18 tails that form a tightly stacked lipid bilayer [108]. In their study, Kulkarni et al. indicated that DSPC with cholesterol and, in general, helper lipids, play an important role in the formulation of siRNA–lipid complexes in LNPs, which is mainly localized on the nanoparticle surface and marginally in the nanoparticle core, internalized together with RNA [109]. Overall, the combination of this phosphatidylcholine-derived lipid with cholesterol promotes the encapsulation of the genetic material in the formulation of LNPs [102]. Kim investigated the possibility of replacing DSCP in LNPs with naturally occurring glycerol derivatives, such as 2-dipalmitoyl-sn-glycero-3-O-4′-(N,N,N-trimethyl)homoserine (DGTS). DTGS is a betaine lipid, derived from plants, characterized by short lipid tails (palmitic acid) in which the zwitterionic headgroup is connected to the glycerol moiety by a branch of hydrocarbons. This type of configuration may alter the LNP formulation in terms of membrane rigidity, a parameter that influences the intracellular delivery efficiency of nucleic acid. In particular, the introduction of DGTS in place of DSPC improves delivery to the liver via intravenous injection but reduces the efficacy of nebulized LNPs for the intranasal delivery of mRNA [110].

The DOPE is composed of a relatively small primary amino headgroup, phosphoethanolamine, and two bulky unsaturated oleoyl tails, responsible for its low melting temperature (about 30 °C), forming a shape like a cone. This lipid geometry forms a more fluid lipid layer and can stabilize the non-bilayer hexagonal II (HII) phase, promoting lipid membrane fusion to the endosomal membrane and/or bilayer disruption [80,105]. For these properties in the LNPs, the presence of DOPE in LNP formulations improves the RNA transfection efficiency compared with DSPC-containing LNPs [30]. Zhang and colleagues investigated the different body distribution of nanoparticles with the same formulation differing only in the presence of DSPC or DOPE, as helper phospholipids. The study reported that LNPs containing the unsaturated phospholipid distribute preferentially in the liver, while those containing DSPC accumulate in the spleen probably for a different interaction with apolipoprotein E (ApoE), stronger for DOPE-LNPs than that with DSPC; suggesting an influence of the structural lipid on LNP biodistribution [80].

To enrich the diversity of phospholipids and, more importantly, to optimize the physical and biological functions of these carriers in SLNs, Liu et al. developed a new type of multi-tailed ionizable phospholipids (iPhos) [111]. These lipids are characterized by a pH-switchable zwitterion headgroup and three hydrophobic tails, which assume a shape like a cone in acidic endosomal environments facilitating hexagonal membrane phase transformation and subsequent RNA release from endosomes. Structure–activity relationships demonstrated that iPhos chain lengths can control the efficacy of mRNA delivery in vivo, formulating, with other helper lipids, multi-component lipid nanoparticles (iPLNPs) for selective organ targeting [112].

In general, phospholipid structure (type of headgroup, chain length, saturation of lipid tail, and lipid tail functional group) plays an important role in increasing the mRNA delivery efficiency of LNPs.

#### 5.2.3. PEG–Lipids

Polyethylene glycol (PEG) is a hydrophilic polymer with many applications in pharmaceutical formulations, and PEGylation has become a gold standard in pharmaceutical nanocarrier modification for developing successful drug delivery systems. PEG–lipid conjugates (PEG–lipids) are lipids in which a hydrophobic alkyl chain is conjugated to a hydrophilic PEG polymer chain; PEG–lipids have been widely used in LNP formulations to deliver anti-cancer drugs, siRNA, and mRNA vaccines. PEG–lipids are present with the smallest mole percentage in the formulation of LNPs, usually not exceeding 1.5 mol%. Their presence, particularly regarding the molar ratio, can influence different NP properties such as particle size, PDI, zeta potential, and stability. LNP size is an important parameter that must be controlled during preparation because it can play a decisive role in their pharmacokinetics, biodistribution, delivery efficiency, and transfection potency [45,113].

Increasing the molar ratio of the PEG–lipid resulted in significantly smaller LNPs, independently of other lipid components. The PEG–lipid is located at the LNP surface, and hence, raising the mol% of the PEG–lipid leads to a higher surface area–volume ratio, and thus, decrease in their particle size.

The type of PEG–lipid concerning structure, chain length, molar mass ratio, and length of the alkyl chain constituting the lipid tail can be crucial in modifying some LNP properties. Which PEG–lipid to choose depends on the therapeutic purpose, target organ and/or cell type, and administration route.

Regarding the PEG chain, a molecular weight of 2000 g/mol, corresponding to about 45 repetition units (PEG 2000), is a good choice for pharmaceutical nanocarriers with a satisfactory elimination half-time and delivery efficiency. PEG with shorter chains is not so performant in terms of preventing protein corona formation and increasing blood circulation time, while PEG with longer chains can have a strong effect on cellular uptake and the endosomal escape process [114]. mRNA-LNP formulations were developed using two short PEG–lipids (C14, dimyristoyl-glycerol) which quickly dissociate from the lipid membrane of LNPs in serum. On the contrary, LNPs with longer lipid chains, such as C18 acyl chain (distearoyl-glycerol), show completely different in vivo behavior with a better anchoring ability on lipid membranes, protecting LNPs and decreasing their interaction with blood proteins [114]. siRNA-loaded LNP formulations containing PEG2000-DMG (1,2-dimyristoyl-rac-glycero-3-methoxypolyethylene glycol-2000) have shorter circulation times and higher in vivo delivery efficacy than formulations containing PEG2000-DSG (1,2-distearoyl-rac-glycero-3-methoxypolyethylene glycol-2000), due to the faster dissociation from the LNPs, which may favor cellular uptake and endosomal escape [115].

During the formation of LNPs, PEG–lipid components arrange themselves with the hydrophilic polymer extended towards the aqueous medium, forming a hydrophilic coating on the surface of the LNPs which can increase their stability reducing aggregation phenomena during storage [24]. The LNP’s ability to deliver their cargo to target cells is directly affected by the PEGylation; in fact, the addition of PEGylated lipid to NP formulations is widely used to increase the in vivo circulation time and prevent rapid clearance from the bloodstream.

For example, in Doxil^®^, which delivers doxorubicin, an anti-cancer drug, the presence of PEG-1,2-distearoyl-sn-glycero-3-phosphorylethanolamine (PEG-DSPE) anchored on the surface of liposomes limits protein binding and clearance, allowing to obtain a formulation with extended circulation time and increasing drug accumulation at the tumor site with a decreasing cardiotoxicity, the main collateral effect, as compared to free doxorubicin [116].

The safety profile of LNPs is also correlated with the lipid components and mRNA molecules. Immunosafety is a key issue in the current research and development of nanomedicines such as LNPs. As already mentioned, the PEG-coating of the NP’s surface reduces the stimulation of the immune system. Conversely, some studies have shown that PEG–lipids could induce hypersensitivity reactions by stimulating the complement system to produce specific antibodies against PEG (anti-PEG). Interestingly, anti-PEG antibodies can be found in individuals who have never received systemic PEGylated therapeutics. This reaction could lead to an increase in NP blood clearance, changing the bioavailability and biodistribution of the drug encapsulated in PEGylated NPs.

**Figure 6:**
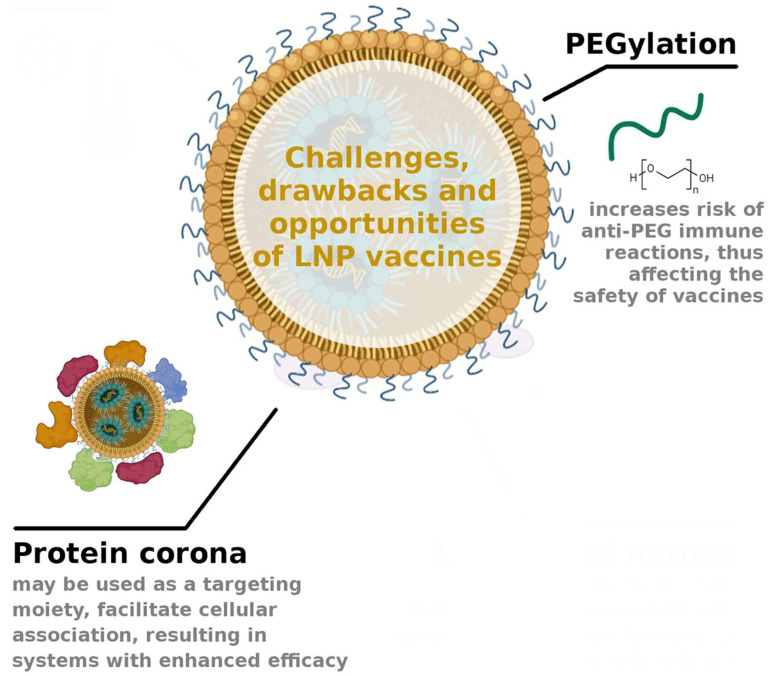
The illustration underscores the primary challenges and drawbacks associated with LNP vaccines. One significant opportunity lies in the ability to modify the protein corona composition, which can help mitigate off-target accumulation and enhance the interaction of LNPs with antigen-presenting cells and dendritic cells. This, in turn, has the potential to significantly improve vaccine efficacy. Reprinted with permission [38].

Other PEG–lipids immunologically induced adverse effects can be accelerated blood clearance (“ABC phenomenon”) observed upon repeated administration, and a hypersensitivity reaction non-IgE-mediated pseudo-allergy, referred to as CARPA (complement activation-related pseudo-allergy), caused by the activation of the complement system, which significantly reduces the efficacy and safety of PEGylated nanocarriers (Figure 6).

Moreover, studies have shown that upon IV administration PEG–lipid with short lipid tails tend to desorb from LNPs, inducing a change in surface structure that allows ApoE adsorption to LNPs, with the formation of a protein corona that affects their endosomal escape and mRNA release. ApoE in blood serum showed specificity for hepatocytes leading to LNP accumulation in the liver as exploited in Onpattro^®^ formulation. In this product, the drug patisiran is delivered using LNPs composed of ionizable lipid (DLin-MC3-DMA), phosphatidylcholine derivate (DSPC), cholesterol, and PEG–lipid (PEG2000-C-DMG). Once the drug is administered into the systemic circulation, the PEG–lipid is replaced by serum proteins, mainly ApoE, which interact with the cholesterol component of the lipid complex and target the drug to the liver [117,118]. Furthermore, PEG–lipids can be functionalized with specific ligands such as folate for siRNA delivery or N-acetylgalactosamine for targeting liver hepatocytes [80].

For instance, the addition of PEG or poloxamine coatings to NPs has been found to reduce complement activation, while dextran coatings increase it. Furthermore, complement activation has been prevented with over 90% efficacy by coating NPs with negative regulatory complement factor H [12]. Similarly, galactose polymer-modified NPs demonstrated lower levels of complement activation compared to glucose-modified nanoparticles, attributed to the adsorption of complement H protein on their surface [13].

### 5.3. Protein Corona

When NPs enter the bloodstream, some biomolecules interact with the LNP surface, resulting in their encapsulation and leading to the formation of a corona. This corona is commonly known as protein corona (PC) due to the predominant presence of plasma proteins [38]. The PC formation is a spontaneous process that occurs rapidly thanks to noncovalent interactions between proteins and the LNP surface [119].

The presence of PC influences many properties of LNPs such as surface charge, hydrophilic and hydrophobic characteristics, stability, targeting ability, aggregation phenomena, toxicity, and efficacy, and, when RNA or DNA are loaded in LNPs, the PC affected also their transfection efficiency [120,121]. Data published on PC-coated LNPs seem to conflict with each other; in fact, many researchers observed a reduction in the targeting ability of nanoparticles after the PC formation [122], an increase in LNP aggregation, a decrease in LNP stability, and the leakage of fluorophores used for LNP tracking in vivo [123] (Figure 6), while other researchers suggested that the PC formation could be favorable to obtain a specific LNP targeting [1].

The PC composition is principally affected by the physico-chemical properties and the biological environment of LNPs; it is also important to underline that the administration of the same LNP formulation to different patients could result in a different interaction of LNP-PC, specific for everyone (depending, as example, on pathological conditions, gender, age, and administration site) [124,125]. The study of the PC seems to be important to obtain a better LNP formulation able to induce the desirable pharmacological effect on each patient [126].

Many studies demonstrated that a careful selection of the technique (or techniques) used to separate LNPs and PC components is one of the key factors in obtaining realistic results. In particular, LNPs resulted in low-dense soft materials that are hardly separable from the serum nanomaterials, including exosomes and HDL [123,127,128]. Until now, the main techniques used to efficiently separate LNPs and PC are size exclusion chromatography (SEC), ultrafiltration, ultracentrifugation, and protein precipitation followed by enzymatic digestion and solid-phase extraction, and flow-field flow fractionation [121,125,127,129,130,131].

On the other hand, the PC composition and formation process were efficiently studied using different techniques and approaches such as liquid chromatography coupled to tandem mass spectrometry (nano LC-MS/MS), sodium dodecyl sulfate-polyacrylamide gel electrophoresis (SDS-PAGE), bicinchoninic acid (BCA) assays, synchrotron small-angle X-ray scattering (SAXS) measurements, and isothermal titration calorimetry (ITC) [121,125,131].

The composition of LNPs and the site of administration affect the PC composition [1,125]; in particular, Qiu and colleagues [1] tested two different LNP formulations, one with amino bond-containing lipids (N-LNPs) and one with ester bond-containing lipids (O-LNPs). The PC of N-LNPs and O-LNPs shared about 800 proteins absorbed on the surface but presented some proteins that specifically bounded only one LNP formulation; in detail, 246 specific proteins are revealed on N-LNPs’ surface including the most abundant albumin, fibrinogen beta chain and fibrinogen gamma chain, while for O-LNPs’ formulation found about 1000 specific proteins with the most abundance of albumin, apolipoprotein E, and complement C1. The study of PC composition by in vitro assays could be very important to define the targeting ability of LNPs before the in vivo administration. The O- and N-LNP formulations developed by Qiu et al. [1] could be used to obtain a specific targeting to the liver and lung, respectively, thanks to their specific interaction with serum proteins. This interesting study suggested that specific formulation competencies are one of the key factors for LNP optimization, underlining the importance of a multidisciplinary collaboration between researchers.

LNP-related PC compositions resulted differently when studies were conducted in vitro or in vivo: Amici et al. [129] demonstrated that only 40% of total protein content was in common between in vitro and in vivo coronas and the in vitro PC resulted in being enriched in higher molecular weight with respect to the in vivo PC. Scientific evidence also demonstrated that in vivo formed LNP-PC presented higher heterogeneity and less amount of complement proteins, which could probably be correlated with a prolonged LNP blood circulation after the administration [129,132]. The in vivo formed PC showed a lower amount of coagulation proteins that are directly involved in the activation of immune system cells by Toll-like receptors; overall, less enrichment in complement and coagulation protein could reduce the particle uptake by immune cells increasing the circulating time [129]. In vivo, PC resulted in being enriched in lipoproteins with respect to the in vitro counterpart, while neither of the two PCs were enriched in immunoglobulins.

Much data on PC composition are published and it is possible to conclude that LC-MS/MS analyses resulted in faster, cleaner, and simpler measurements to obtain information from complex biological samples but the critical aspect is the nature of considered PC; only a few data are reported on in vivo formed LNP-PC and further research will be needed to understand the effect of many factors, such as the administration route, the LNPs’ compositions, and the presence of any pathologies, on PC composition, and consequently, on the fate of LNPs once administered.

## 6. Conclusions

The survey of the literature shows that LNPs have great promises in delivering RNA-based therapeutics, but they still require studies that clarify how nanocarrier properties, including size, shape, and surface chemistry, can direct the particles into target sub-cellular compartments and cause immunomodulatory or immunotoxicity effects.

The immune system can recognize LNPs as foreign, leading to an immune response that limits their therapeutic efficacy or can support anti-infective and anti-cancer applications. To better understand the interaction between mRNA-LNPs and the immune system, of paramount importance is the knowledge of cytokines/chemokines, complement, and anti-drug antibodies involved, as more reliable indicators. These can predict potential side effects more accurately. The availability of in vitro and in vivo models for assessing mRNA-LNP therapeutics’ efficiency and safety in early research and development phases still requires some improvement.

In this review, LNP advantages and their interaction with the immune system, which is not always a favorable characteristic for their use, have been highlighted. In non-immunotherapy applications, it is crucial to fine-tune the mRNA-LNP components to minimize immune activation and increase safety. Therefore, it is necessary to understand the basic pharmacodynamics and pharmacokinetics of mRNA-LNP non-immunotherapy drug candidates and their interaction with the host immune system. The immune response can lead to side effects, but in the case of prophylactic therapy, such as vaccines or oncologic therapy, an immune response can be beneficial. In most immunotherapy applications (infectious disease and cancer vaccines), a boost to the immune system based on natural recognition of synthetic mRNAs and LNP components that mimic a viral attack may be essential.

## Figures and Tables

**Table 1:** Advantages and limits correlated with the use of cationic and ionizable lipids into LNP formulations.

		Advantages	Limits
**Ionizable lipids**	MC3 (Onpattro^®^) ALC-0315 (Comirnaty^®^) SM-102 (SpikeVax^®^) KC2 DODAP DODMA	Positive charge at low pH → high interaction with RNA/DNA [85] Neutral charge at physiological pH → no toxicity [62,85] Positive charge at acidic endosomal pH → effective RNA/DNA release [85] Improvement of spleen targeting [64] FDA/EMA-approved	Complex, time-consuming, costly synthesis process [86] Not completely biodegradable [85]
**Cationic lipids**	DDA DOTAP DOTMA	High cell interaction [62] Lysosomal rupture [62] High cargo release [62] Transfection efficiency improvement Lung targeting improvement [64] Ionic interaction with antigens → high cell-mediate and humoral immune response [67,68,74,75]	Cell membrane disruption [20] Toxicity [20,65] Induction of pro-inflammatory cytokine release [29,77]

**Table 2:** Schematic summary of the techniques used to measure/determine physico-chemical properties of LNPs; the biological in vitro and in vivo effects of each considered LNP characteristic were reported.

Physico-Chemical Properties of LNPs	Measurement Techniques	In Vitro and In Vivo Effects [References]
**Particle size distribution,** **polydispersity index**	Dynamic light scattering (DLS) Size exclusion chromatography (SEC) Field flow fractionation (FFF) Tunable resistive pulse sensing (TRPS)	Internalization [32,33] Circulation half-lives and In vivo distribution [35] In vitro transfection efficacy [36] Immune response [14,36,39,40,41,42,43,44]
**Shape**	Transmission electron microscopy (TEM) Scanning electron microscope (SEM)	Cellular uptake [48,53,59] Circulation half-lives [14,49,50,51] Immunogenicity [55]
**Elasticity**	Transmission electron microscopy (TEM) Cryo-electron microscope (CryoEM)	Clearance mechanisms [56] Interaction with immune cells [58] Cellular uptake [59] Systemic circulation lifetime [60]
**Surface chemistry** **and charge**	Dynamic light scattering (DLS), Electrophoretic light scattering (ELS)	Cellular uptake [62] Nitrogen to phosphorus ratio (N/P ratio) [63] Organ targeting [64] Toxicity [65,66] Immunogenic response [67,71,72,73]

## Data Availability

No new data were created, or analyzed in this study. Data sharing is not applicable to this article.
