# Correction: Catenacci et al. Effect of Lipid Nanoparticle Physico-Chemical Properties and Composition on Their Interaction with the Immune System. *Pharmaceutics* 2024, *16*, 1521

**DOI:** 10.3390/pharmaceutics18040412

**Published:** 2026-03-28

**Authors:** Laura Catenacci, Rachele Rossi, Francesca Sechi, Daniela Buonocore, Milena Sorrenti, Sara Perteghella, Marco Peviani, Maria Cristina Bonferoni

**Affiliations:** 1 Department of Drug Sciences, University of Pavia, 27100 Pavia, Italy; laura.catenacci@unipv.it (L.C.); rachele.rossi01@universitadipavia.it (R.R.); francesca.sechi01@universitadipavia.it (F.S.); milena.sorrenti@unipv.it (M.S.); mariacristina.bonferoni@unipv.it (M.C.B.); 2 Department of Biology and Biotechnology “L. Spallanzani”, University of Pavia, 27100 Pavia, Italy; daniela.buonocore@unipv.it

**Error in Table**

In the original publication [1], there was a mistake in Table 1 as published. DODMA has been classified as ‘Cationic lipids’ and should be reclassified under ‘Ionizable lipids,’ as its surface charge is pH-dependent. Also, DOPE should not be classified as a ‘Cationic lipids’ and has been removed from Table 1. Additionally, ALC-0159 should not be classified as an ‘Ionizable lipid’ and has been removed from Table 1. The corrected Table 1 appears below. The authors state that the scientific conclusions are unaffected. This correction was approved by the Academic Editor. The original publication has also been updated.

The authors state that the scientific conclusions are unaffected. This correction was approved by the Academic Editor. The original publication has also been updated.

## Figures and Tables

**Table 1:** Advantages and limits correlated with the use of cationic and ionizable lipids into LNP formulations.

		Advantages	Limits
**Ionizable lipids**	MC3 (Onpattro^®^) ALC-0315 (Comirnaty^®^) SM-102 (SpikeVax^®^) KC2 DODAP DODMA	Positive charge at low pH → high interaction with RNA/DNA [85] Neutral charge at physiological pH → no toxicity [62,85] Positive charge at acidic endosomal pH → effective RNA/DNA release [85] Improvement of spleen targeting [64] FDA/EMA-approved	Complex, time-consuming, costly synthesis process [86] Not completely biodegradable [85]
**Cationic lipids**	DDA DOTAP DOTMA	High cell interaction [62] Lysosomal rupture [62] High cargo release [62] Transfection efficiency improvement Lung targeting improvement [64] Ionic interaction with antigens → high cell-mediate and humoral immune response [67,68,74,75]	Cell membrane disruption [20] Toxicity [20,65] Induction of pro-inflammatory cytokine release [29,77]

## References

[1] Catenacci L., Rossi R., Sechi F., Buonocore D., Sorrenti M., Perteghella S., Peviani M., Bonferoni M.C. (2024). Effect of Lipid Nanoparticle Physico-Chemical Properties and Composition on Their Interaction with the Immune System. Pharmaceutics.

